# Hippocampal Brain Volume Is Associated with Faster Facial Emotion Identification in Older Adults: Preliminary Results

**DOI:** 10.3389/fnagi.2016.00203

**Published:** 2016-08-25

**Authors:** Sarah M. Szymkowicz, Jonas Persson, Tian Lin, Håkan Fischer, Natalie C. Ebner

**Affiliations:** ^1^Department of Clinical and Health Psychology, University of FloridaGainesville, FL, USA; ^2^Aging Research Center, Karolinska InstituteStockholm, Sweden; ^3^Department of Psychology, University of FloridaGainesville, FL, USA; ^4^Department of Psychology, Stockholm UniversityStockholm, Sweden; ^5^Department of Aging and Geriatric Research, University of FloridaGainesville, FL, USA

**Keywords:** hippocampus, brain volume, faces, emotion identification, structural MRI

## Abstract

Quick correct identification of facial emotions is highly relevant for successful social interactions. Research suggests that older, compared to young, adults experience increased difficulty with face and emotion processing skills. While functional neuroimaging studies suggest age differences in neural processing of faces and emotions, evidence about age-associated structural brain changes and their involvement in face and emotion processing is scarce. Using structural magnetic resonance imaging (MRI), this study investigated the extent to which volumes of frontal and temporal brain structures were related to reaction time in accurate identification of facial emotions in 30 young and 30 older adults. Volumetric segmentation was performed using FreeSurfer and gray matter volumes from frontal and temporal regions were extracted. Analysis of covariances (ANCOVAs) models with response time (RT) as the dependent variable and age group and regional volume, and their interaction, as independent variables were conducted, controlling for total intracranial volume (ICV). Results indicated that, in older adults, larger hippocampal volumes were associated with faster correct facial emotion identification. These preliminary observations suggest that greater volume in brain regions associated with face and emotion processing contributes to improved facial emotion identification performance in aging.

## Introduction

The ability to quickly recognize faces and their emotional expressions are important skills that we develop early in life. These skills remain relevant across the entire lifespan and are important to our everyday functioning, particularly with respect to social interactions. Similar to other cognitive abilities, research indicates that facial emotion identification abilities decline with age. Older adults typically experience greater difficulty in reading facial cues, particularly for negative facial emotions (Isaacowitz et al., 2007; Ruffman et al., 2008, 2012; Ebner and Fischer, 2014). These difficulties associated with recognizing emotion displayed in faces can negatively impact overall emotional and social functioning in the elderly (Isaacowitz et al., 2007) and, thus, are of critical concern.

Previous studies that showed age-related difficulties in facial emotion identification have examined associations of this deficit with age differences in functional brain activation (Fischer et al., 2005, 2010; Gao et al., 2009; Lee et al., 2011; Ebner et al., 2012). It is proposed that multiple brain regions work together in order for face processing, independent of emotional valence, to occur (for review see Ruffman et al., 2008). The processing of emotional facial stimuli involves the amygdala, the medial prefrontal, and anterior cingulate cortices, insula and hippocampus, which are shown to be active in both young and older adults (Iidaka et al., 2002; Fusar-Poli et al., 2009). However, there are age differences in neural recruitment during processing of emotional faces (Keightley et al., 2007). This research suggests that older adults have a more widely distributed network for processing negative and neutral facial expressions, including both frontal and temporal regions. In contrast, young adults show a more widely distributed network for processing happy facial expressions. Additional evidence suggests age-related reduced activity in medial temporal (i.e., amygdala, parahippocampal gyrus) and parieto-occipital regions during emotional face processing (Iidaka et al., 2002; Ebner et al., 2012) and that older adults show greater temporal activity (i.e., hippocampus, fusiform gyrus) compared to young adults during these tasks (Fusar-Poli et al., 2009).

A considerable amount of research in facial emotion processing relies on functional neuroimaging studies, which allow brain activity to be linked with function (e.g., behavioral performance). However, functional activation is likely dependent on structural integrity, which has not been the focus of studies in this field of research. Gray matter brain volume is comprised of cortical surface area and cortical thickness. Cortical surface area reflects the number of cell columns, while cortical thickness is a measure of the number and size of cells within the column, their packing density, and the number of synaptic connections (Kabani et al., 2001; Eickhoff et al., 2005). The neuroanatomical volumetric basis for cognitive aging has been well-researched (Raz and Kennedy, 2009; Salthouse, 2011); however, close to nothing is known about the neuroanatomical basis for various social processing domains. In this study, we argue that structural intactness (i.e., as reflective in gray matter volume) of anterior cingulate and insula cortices, fusiform gyrus, amygdala and hippocampus are associated with preserved facial emotion processing and will particularly facilitate facial emotion identification in aging. This prediction was based on evidence that older adults’ difficulties with social processing are due to decline in structural brain regions supporting social–emotional circuits (Phillips et al., 2014).

In general, aging is associated with overall decline in total gray matter volume (Raz et al., 1997, 2010; Resnick et al., 2003; Fjell and Walhovd, 2010). The largest atrophic changes are seen in the frontal and temporal cortices (Hedden and Gabrieli, 2004; Raz and Kennedy, 2009), regions known to be involved in facial emotion processing, though this degradation does not occur in a uniform manner. There is also evidence of age-related decline in the insula and fusiform gyrus. Decline in these regions, however, is less pronounced compared to decline in other brain regions (Good et al., 2001; Allen et al., 2005; Raz et al., 2005, 2010; Fjell and Walhovd, 2010; Persson et al., 2014).

In young adults, larger amygdala volume has been shown to be associated with decreased accuracy for recognizing fearful faces and increased likelihood of misinterpreting fear as surprise (Zhao et al., 2013). In adults with traumatic brain injury, difficulties with the recognition of facial affect was related to reduced white matter integrity, as well as reduced gray matter volumes in temporo-occipital regions (Genova et al., 2015). These studies suggest that there is a relationship between brain structure and facial emotion processing in young and brain-injured adults. Some first evidence of a possible relationship between brain structure and performance in aging comes from Williams et al. (2006), who found that smaller medial prefrontal cortex volumes were associated with decreased accuracy for recognizing fearful faces. While these previous studies hint at a relationship between brain structure and behavioral function, a systematic examination and adult age comparison of frontal and temporal gray matter volume in their effect on facial emotion identification performance are still warranted.

The present study examined the effects of age and brain volume on speed of facial emotion identification. In particular, our primary aim was to determine the extent to which brain volume in fronto-temporal regions (i.e., anterior cingulate and insula cortices, fusiform gyrus, amygdala, and hippocampus) was associated with performance variability in the speed of facial emotion identification in young and older adults. We expected that greater volume in regions with a known role in facial emotional processing would predict better behavioral performance, as reflected in faster responses for accurate identification of facial emotions (*Hypothesis 1*). Given previous evidence for age-related atrophy in these target regions, we also expected that the association between brain volume and behavioral performance would be more pronounced in older, compared to young, adults (*Hypothesis 2)*.

## Materials and Methods

### Participants

Thirty young and 30 older adults were recruited from the local community by means of an advertisement in a local newspaper. Table 1 presents descriptive information for demographic, cognitive, and affective measures. All participants were right-handed native Swedish speakers with normal or corrected-to-normal vision and were in good self-reported health, with no known history of neurological or cardiovascular disorders, and none were taking psychotropic medications. For the older participants, a radiologist screened the T1- and T2-weighted structural scans to ensure absence of abnormal atrophy and/or lesions. Each person provided written informed consent after the experimental procedures were explained in accordance with the Declaration of Helsinki. The study and data analysis were approved by the Ethics Committee in Stockholm and by the University of Florida. Participants were financially compensated.

**Table 1:** **Means (M), standard deviations (SD), and age differences for demographic, cognitive, and affective measures**.

Measures	Young participants		Older participants		Age differences
	*M*	*SD*	*M*	*SD*	
**Demographics**
Age	25.13	3.38	68.27	2.52	–
Sex (% M/F)	47/53	–	43/57	–	*X^2^_(1, N = 60)_* = 0.67, *p* = 0.795
Education	14.80	2.12	13.97	2.90	*F*_(1,58)_ = 1.62, *p* = 0.209, $\eta_{p}^{2}$ = 0.03
**Cognitive functioning**
MMSE	29.35	0.55	28.90	0.94	*F*_(1,56)_ = 4.91, *p* = 0.031, $\eta_{p}^{2}$ = 0.08*
LCT	11.02	2.06	8.36	1.90	*F*_(1,57)_ = 26.48, *p* < 0.001, $\eta_{p}^{2}$ = 0.32***
FWRT	10.03	2.34	7.26	1.85	*F*_(1,57)_ = 25.09, *p* < 0.001, $\eta_{p}^{2}$ = 0.31***
2-Back	8.44	1.38	6.30	1.98	*F*_(1,55)_ = 22.58, *p* < 0.001, $\eta_{p}^{2}$ = 0.29***
SST	22.57	3.68	26.50	2.08	*F*_(1,56)_ = 24.59, *p* < 0.001, $\eta_{p}^{2}$ = 0.31***
VFT	15.05	4.97	15.45	4.52	*F*_(1,56)_ = 0.10, *p* = 0.752, $\eta_{p}^{2}$ = 0.00
**Affective functioning**
GDS	1.37	1.63	1.14	1.65	*F*_(1,56)_ = 0.27, *p* = 0.605, $\eta_{p}^{2}$ = 0.01
STAI-S	30.52	5.35	28.59	6.75	*F*_(1,56)_ = 1.46, *p* = 0.232, $\eta_{p}^{2}$ = 0.03

Note: M, male; F, female; MMSE, Mini-Mental Status Examination (cognitive screening); LCT, Letter Comparison Task (processing speed); FWRT, Free Word Recall Task (episodic memory); 2-Back, 2-Back Digits Task (working memory); SST, Swedish Synonym Task (vocabulary); VFT, Verbal Fluency Task (verbal fluency); GDS, Geriatric Depression Scale (depression); STAI-S, State-Trait Anxiety Inventory, State version (state anxiety). There were missing data for questionnaires and covariates for one older woman; missing data for 2-Back for one young man and one older woman; missing data for STAI-S for one young man. Outlier data were removed for the MMSE (one young woman), SST (one older woman), VFT (one older female), GDS (one older female), and STAI-S (one older man). *p < 0.05, ***p < 0.001.

### Procedures and Measures

Participants came in for two sessions. During the first session, they filled out several paper-and-pencil questionnaires for sample descriptive purposes that included demographic questions, the Mini-Mental State Examination (MMSE; cognitive screening; Folstein et al., 1975), a Swedish version of the Geriatric Depression Scale (GDS; Brink et al., 1982; Gottfries, 1997), and the state version of the State-Trait Anxiety Inventory (Spielberger et al., 1983). Several computer-based tasks were also completed, including the Letter Comparison Task (LCT; processing speed; Salthouse and Babcock, 1991), a verbal episodic memory free recall task, a 2-Back working memory task (Kirchner, 1958), a Swedish Synonym Task (SST; vocabulary; Dureman, 1960) and a Verbal Fluency Task (VFT; verbal fluency to letters F and A; Lezak, 1995).

**Figure 1:**
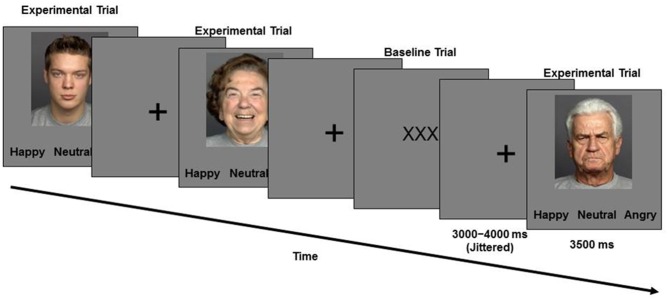
**Schematic of the *Facial Emotion Identification* Paradigm**.

During the second session, approximately a week later, participants performed the facial emotion identification task while functional images of their brain were taken. Functional magnetic resonance imaging (fMRI) methods and results are the focus of reports elsewhere (Ebner et al., 2012, 2013). In this task (Figure 1), a set of 96 young and older face images with equal numbers of happy, angry, and neutral expressions were presented. Participants saw faces one at a time and were asked to indicate via a button press as quickly and accurately as possible whether the displayed face showed a happy, angry or a neutral expression. The presentation order of face identities was the same for each participant, with facial expressions counterbalanced across participants (each participant only saw each face with one expression; for details see Ebner et al., 2012, 2013).

Face images were taken from the FACES database (Ebner et al., 2010). Stimulus presentation and behavioral data were collected using E-Prime software (Schneider et al., 2002).

### Neuroimaging Acquisition

Magnetic resonance imaging (MRI) data were collected on a 3T Siemens Magnetom Trio Tim Scanner at Huddinge Hospital in Stockholm, Sweden, using a 32-channel head coil. High resolution, T1-weighted MPRAGE anatomical scans were collected using the following parameters: TR = 1900 ms, TE = 2.52 ms, 176 slices acquired in a sagittal orientation, flip angle = 9°, FOV = 256 mm, 1 mm cubic resolution. Task-related stimuli were presented on a computer screen viewed by participants via a reflective mirror mounted on the head coil and responses were collected using a scanner-compatible response pad. To minimize noise while in the scanner, participants were given headphones and earplugs. Head movement was minimized via cushions positioned inside the head coil.

### Volumetric Measurement

We used Freesurfer image analysis suite (version 5.1^1^) for volumetric segmentation, cortical surface reconstruction, and parcellation to quantify the brain volumes of interest (for technical details of these procedures, see Dale et al., 1999; Fischl et al., 2002; Han et al., 2006). These procedures ran automatically, but required supervision for the accuracy of spatial registration and tissue segmentation. Using this method, we obtained left and right volumes from the anterior cingulate and insula cortices, fusiform gyrus, amygdala and hippocampus from the T1-weighted images, and an estimate of total intracranial volume (ICV). Caudal and rostral anterior cingulate cortex values were summed together to obtain total volumetric values for those regions. Right and left volumes were averaged to obtain a total bilateral volumetric value for each region of interest (ROI), as our hypotheses were not hemisphere specific. Left and right brain region values were averaged together, as the general pattern of results was similar across the two hemispheres.

### Statistical Analyses

Statistical analyses were conducted using SPSS 22.0 software (Armonk, NY, USA). Prior to statistical analysis, we screened data for outliers by computing standardized *z*-scores for all dependent and independent variables. Outliers were identified as *z* > ±3 standard deviations within each age group and removed from analyses (see Tables 1, 3 notes for details). Behavioral raw scores for speed of correct facial emotion identification served as the outcome variable. One design weakness of previous studies has been that they presented only one positive along with several different negative facial emotions. This makes identification of positive and negative facial emotions qualitatively different tasks. To overcome this weakness, we presented only one positive, one neutral and one negative facial expression (as described above under “Procedures and Measures” Section. This resulted in high accuracy performance (near ceiling) for both age groups and thus there was little variance to predict for this outcome variable. Therefore, accuracy was not considered in the current investigation, though it is reported in Table 3 for descriptive purposes. To address our primary aim, analyses for the facial emotion identification task investigated response time (RT) collapsed across all emotional faces and across the age of the faces. *Post hoc*, we also explored emotion-specific and age-of-face specific effects as reported below.

We conducted independent samples *t*-tests (and a chi-square analysis for sex) to examine age differences in demographic, cognitive, and affective measures (Table 1). Correlational analyses were run to determine the relationship between cognitive measures assessed in the study (Table 2) and accuracy as well as RT in the facial emotion identification task (for each age group separately and across the total sample). Analysis of covariance (ANCOVAs) with RT as the dependent variable, age group (young vs. older) as the categorical predictor variable, and ROI volume as continuous predictor variable tested *Hypothesis 1* and *2*. We also entered the age group × ROI volume interaction in the models and controlled for ICV. Initially, sex and education were entered as additional covariates of non-interest, but were later removed due to lack of statistical significance. We applied a statistical significance threshold of *α* < 0.050 for our directional hypotheses. Effect sizes are reported as partial eta-squared ($\eta_{p}^{2}$), for which 0.01 indicates a small effect, 0.06 a medium effect and 0.14 a large effect (Cohen, 1969).

**Table 2:** **Bivariate correlations between cognitive measures**.

	MMSE	LCT	FWRT	2-Back	SST	VFT	RT faces
MMSE	–	–	–	–	–	–	–
LCT	−0.00	–	–	–	–	–	–
FWRT	0.20	0.40**	–	–	–	–	–
2-Back	0.27*	0.30*	0.40**	–	–	–	–
SST	−0.05	−0.17	−0.14	−0.22	–	–	–
VFT	0.19	0.11	0.21	0.18	0.39**	–	–
RT faces	−0.18	−0.24	−0.34**	−0.28*	−0.02	−0.07	–
Accuracy faces	−0.02	−0.07	0.24	−0.02	−0.05	−0.11	−0.38**

Note: MMSE, Mini-Mental Status Examination (cognitive screening); LCT, Letter Comparison Task (processing speed); FWRT, Free Word Recall Task (episodic memory); 2-Back, 2-Back Digits Task (working memory); SST, Swedish Synonym Task (vocabulary); VFT, Verbal Fluency Task (verbal fluency); RT, response time. *p < 0.05, **p < 0.01.

## Results

### Sample Characteristics

As shown in Table 1, there were no age differences in sex distribution, years of education, verbal fluency, depression, or state anxiety. In line with other representative samples in the literature, young participants scored better on the cognitive screening, processing speed, episodic memory and working memory measures, while older participants scored better on the vocabulary measure area. Table 2 depicts the relationship between cognitive measures for the entire sample. Accuracy and RT were moderate-to-strongly correlated for the older participants (*r*_(28)_ = −0.54, *p* = 0.002) and in the total sample (*r*_(57)_ = −0.38, *p* = 0.003) and were trend-wise correlated for the young participants (*r*_(27)_ = −0.36, *p* = 0.056).

### Age Differences in Brain Volumes and Behavioral Performance

Table 3 gives a full characterization of age differences in the brain volumes and the behavioral performance. In sum, compared to young participants, older participants had smaller volumes of the anterior cingulate cortex, amygdala and hippocampus and were slower to identify facial emotional expressions. There were no significant age differences in volume for the insula and the fusiform gyrus.

**Table 3:** **Means (M), standard deviations (SD), and age differences for brain volumes (in mm^3^) and response time (RT) and accuracy for facial emotion identification**.

	Young participants		Older participants		Age differences
M	SD	M	SD
**Brain volumes**
Anterior cingulate cortex	5752	594	5292	611	F_(1,58)_ = 8.73, p = 0.005, $\eta_{p}^{2}$ = 0.13**
Insula	9326	1146	9366	776	F_(1,58)_ = 0.03, p = 0.874, $\eta_{p}^{2}$ = 0.00
Fusiform gyrus	7041	680	6720	871	F_(1,58)_ = 2.53, p = 0.117, $\eta_{p}^{2}$ = 0.04
Amygdala	1747	180	1585	232	F_(1,57)_ = 9.02, p = 0.004, $\eta_{p}^{2}$ = 0.14**
Hippocampus	4444	396	4053	384	F_(1,57)_ = 14.82, p < 0.001, $\eta_{p}^{2}$ = 0.21***
**Facial emotion identification**	
Response time (in ms)	1298	218	1422	208	F_(1,58)_ = 5.12, p = 0.027, $\eta_{p}^{2}$ = 0.08*
Accuracy (%)	94	7	95	5	F_(1,57)_ = 0.39, p = 0.534, $\eta_{p}^{2}$ = 0.01

Note: Outlier data were removed for accuracy for one young man. Outlier data for the amygdala and hippocampus were removed for one young man. The pattern of results without outlier deletion was comparable. *p < 0.05, **p < 0.01, ***p < 0.001.

### Effects of Brain Volume on Facial Emotion Identification in Young and Older Participants

**Figure 2:**
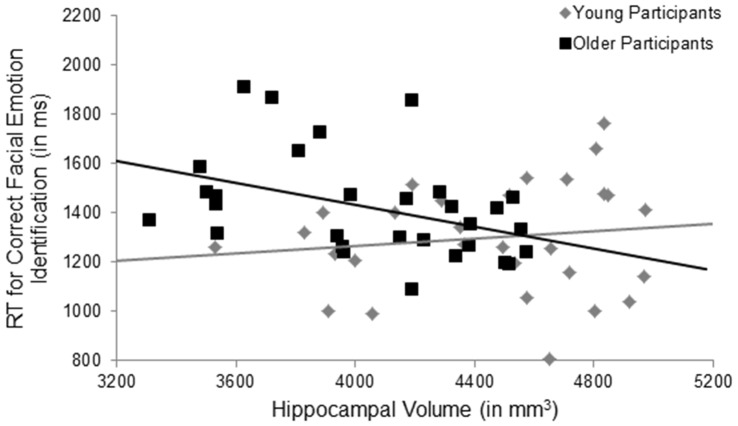
**Significant results for the age × hippocampal volume interaction for facial emotion identification.** Larger hippocampus volumes were significantly associated with faster response times during correct facial emotion identification in older, but not young, participants, *p* = 0.040, uncorrected. RT, response time; ms, milliseconds; mm, millimeters.

*Hypothesis 1* stating that larger volume in brain regions associated with facial emotion processing predicted faster RT in correct facial emotion identification in the total sample was not supported. However, in support of *Hypothesis 2*, there was a significant age × ROI volume interaction in the hippocampus (*F*_(1,54)_ = 4.41, *p* = 0.040, $\eta_{p}^{2}$ = 0.08, uncorrected), such that larger hippocampus volume was significantly associated with faster RTs during correct facial emotion identification in older, but not young, participants (Figure 2). None of the other main effects or interactions was significant (*p*’s > 0.05; Table 4).

**Table 4:** **Effects of age and brain volume on RT (in ms) for correct facial emotional identification (adjusted for total intracranial volume, ICV)**.

		Age			Volume			Age × Volume		
	df	F	p	$\eta_{p}^{2}$	F	p	$\eta_{p}^{2}$	F	p	$\eta_{p}^{2}$
Anterior cingulate cortex	1.55	0.45	0.51	0.01	2.47	0.12	0.04	0.73	0.40	0.01
Insula	1.55	0.12	0.73	0.00	2.32	0.13	0.04	0.01	0.91	0.00
Fusiform gyrus	1.55	0.49	0.49	0.01	0.13	0.72	0.00	0.23	0.64	0.00
Amygdala	1.54	2.25	0.14	0.04	0.12	0.73	0.00	1.68	0.20	0.03
Hippocampus	1.54	5.00	0.03*	0.09	1.40	0.24	0.03	4.41	0.04*	0.08

Note: df, degrees of freedom for each F-test. Outlier data from the amygdala and hippocampus were removed for one young man. The pattern of results without outlier deletion was comparable. *p < 0.05, uncorrected.

### *Post hoc* Analyses

Even though we did not have specific hypotheses pertaining to the effects of brain volume on RT for happy, neutral and angry facial emotions, respectively, or as a function of the age of the face, based on previous research on behavioral age differences in facial emotion identification speed between young and older adults for different emotional expressions (e.g., Mather and Knight, 2006) and a robust literature on own-age effects in processing faces (Rhodes and Anastasi, 2012), we explored effects of facial emotion and age of face in *post hoc* analyses. These analyses parsing apart the effects of individual facial emotions and age-of-face were conducted using repeated-measure ANCOVA analyses, with age group (young vs. older) as the between-subjects factor, ROI volume as the continuous predictor, and either facial expression (happy vs. neutral vs. angry) or age of face (young vs. older) as within-subject factors, controlling for total ICV.

The main effect of facial emotion was not significant. The facial emotion × anterior cingulate cortex interaction was significant (*p* = 0.048, uncorrected), such that across all three emotions, those with larger anterior cingulate volumes had faster RTs. None of the other two-way or three-way interactions were significant.

As the analyses for the individual facial emotions were largely non-significant, the analyses considering age of faces as additional predictor were collapsed across facial emotions. The main effect of age of face was significant (*F*_(1,54)_ = 5.05, *p* = 0.029, $\eta_{p}^{2}$ = 0.09), such that participants were faster to respond to young (*M* = 1298, SD = 212) compared to older (*M* = 1422, *SD* = 237) faces. However, none of the two-way or three-way interactions were significant.

To determine specificity of our obtained effect (i.e., age × hippocampus volume predicted RT for facial emotion identification), *post hoc* analyses investigating whether hippocampal volume predicted cognitive performance were conducted using identical ANCOVA models as described under “Statistical Analyses” Section, with the outcome RT variable being replaced by the other cognitive measures (as listed in Table 2). As seen in Table 5, the age × hippocampus interaction was not significant for any of the cognitive measures (*p*’s > 0.05).

**Table 5:** ***Post hoc* analyses on the effects of age and hippocampal volume on cognitive measures (adjusted for total ICV)**.

		Age			Volume			Age × Volume		
	df	F	p	$\eta_{p}^{2}$	F	p	$\eta_{p}^{2}$	F	p	$\eta_{p}^{2}$
MMSE	1.53	0.01	0.93	0.00	2.47	0.12	0.05	0.03	0.87	0.00
LCT	1.53	0.08	0.78	0.00	0.08	0.77	0.00	0.02	0.89	0.00
FWRT	1.53	4.77	0.03*	0.08	0.23	0.64	0.00	2.96	0.09	0.05
2-Back	1.51	3.99	0.05	0.07	0.00	0.96	0.00	2.33	0.13	0.04
SST	1.53	1.32	0.26	0.02	1.19	0.28	0.02	0.49	0.49	0.01
VFT	1.53	2.82	0.10	0.05	1.06	0.31	0.02	2.69	0.11	0.05

Note: df, degrees of freedom for each F-test; MMSE, Mini-Mental Status Examination (cognitive screening); LCT, Letter Comparison Task (processing speed); FWRT, Free Word Recall Task (episodic memory); 2-Back, 2-Back Digits Task (working memory); SST, Swedish Synonym Task (vocabulary); VFT, Verbal Fluency Task (verbal fluency). *p < 0.05, uncorrected.

**Figure 3:**
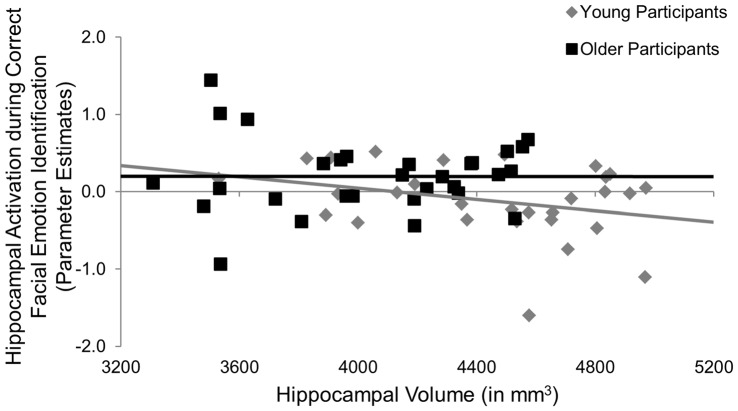
***Post hoc* results for the effects of age × hippocampal volume on hippocampal activation during the facial emotion identification task.** Larger hippocampus volumes were significantly associated with less hippocampal activation in young, but not older, participants, p = 0.040. mm, millimeters.

Participants underwent fMRI while engaging in the facial emotion identification task. Therefore we were able to explore the structure-function-behavior relationship for the significant finding in hippocampus. Hippocampal activation was extracted via the MarsBaR ROI toolbox (Brett et al., 2002) for SPM12 (Wellcome Trust Centre for Neuroimaging, London, UK). Using ANCOVA models, hippocampal activation was the dependent variable, age group was entered as a categorical predictor and hippocampal volume and RT collapsed across all emotional faces were continuous predictor variables, controlling for ICV. We also entered the age group × volume, age group × RT, and age group × volume × RT interactions into the model. As seen in Table 6, the age group × hippocampal volume interaction was significant (*F*_(1,50)_ = 4.47, *p* = 0.040, $\eta_{p}^{2}$ = 0.08), such that larger hippocampus volumes were significantly associated with less hippocampal activation in young, but not older, participants (Figure 3). None of the main effects or any of the other interactions was significant.

**Table 6:** ***Post hoc* analysis of the effects of age, hippocampal volume (mm^3^) and RT (in ms) for correct facial emotional identification on hippocampal activation (adjusted for total ICV)**.

	Hippocampal activation		
	F	p	$\eta_{p}^{2}$
ICV	0.37	0.55	0.01
Age	–	–	–
Hippocampal volume	0.23	0.63	0.01
Response time	0.10	0.76	0.00
Age × hippocampal volume	4.47	0.04*	0.08
Age × response time	4.02	0.05	0.07
Hippocampal volume × response time	0.22	0.64	0.00
Age × Hippocampal volume × response time	3.94	0.05	0.07

Note: mm, millimeters; ms, milliseconds; ICV, intracranial volume. *p < 0.05.

## Discussion

The central aim of the present study was to determine the effects of frontal and temporal brain volume on speed of accurate identification of facial emotions in young and older adults. We report novel evidence supporting a link between brain volume and behavioral performance on a face emotion identification task in aging. Older participants had less gray matter volume in the anterior cingulate cortex, amygdala and hippocampus compared to that of young participants. Importantly, larger hippocampus volumes in older adults were associated with faster RTs, supporting a beneficial relationship between brain structure and facial emotion identification abilities in aging.

It has been suggested that individual differences within the brain allow some people to better deal than others with age-related and/or pathological brain changes (i.e., brain reserve; Stern, 2009). Larger brain volumes, and presumably more neurons and synaptic connections, may slow down age-related cognitive decline. In older adults, larger brain structures have been associated with better cognitive performance (Rushton and Ankney, 2009; Kaup et al., 2011; Royle et al., 2013). In line with these studies, our data suggest that larger brain volumes in task-specific regions of the brain are predictive of better cognitive functioning in older adults and lend support to the brain reserve hypothesis. Support for the brain reserve hypothesis in the literature comes from the relationship between educational attainment and delayed onset of pathological cognitive aging (Stern, 2009). However, in our study, education was not a significant predictor.

Notably, our results do not suggest that having larger brain volume in general is predictive of better overall cognitive performance. Rather, our results suggest that older adults with larger brain volumes in task-relevant regions have better cognitive performance on those tasks. Specifically, our study demonstrated that older, but not young, adults with larger hippocampal volumes are faster with the processing of facial emotions. In addition, these effects were limited to facial emotion processing and did not hold for other, face and emotion unrelated, cognitive processes.

There is some indication that age-related changes in brain structure may influence the relationship between brain activity and behavior, suggesting a complex relationship between these processes. For example, Rajah et al. (2011) found a positive relationship between middle frontal gyrus volume, brain activity of the episodic retrieval network, and memory performance in young adults. For older adults, larger volume was related to less activity in parahippocampal and anterior cingulate cortices, which predicted better memory performances. This suggests that older adults with larger brain volumes may be better able to compensate for age-related structural changes by modifying activity in other brain regions. Likewise, Kalpouzos et al. (2012) found that brain activity in older adults during episodic encoding and retrieval was driven by gray-matter atrophy. This suggests that structural brain changes may account for some of the brain activity differences seen between young and older adults. Similar complex relationships have been reported for the influence of age-related structural change on the relationship between brain activity and identification of facial emotions. In older adults compared to young and middle-aged adults, smaller medial prefrontal cortex volumes predicted decline in fear recognition, but not functional brain activity changes in this region (Williams et al., 2006). It may be that brain activity in other regions important for emotion processing (i.e., basal ganglia) compensates for the age-related structural change in the prefrontal cortex.

Our *post hoc* analyses did not evidence a structure-function-behavior relationship. While the structural integrity of the hippocampus may be important for behavioral performance, it did not predict hippocampal activation during the task. There is evidence that more intact white matter has been shown to influence brain activation and this relationship predicted task performance (Burzynska et al., 2013). Likewise, Thomas et al. (2008) showed that age-related structural connectivity differences between the right temporal and frontal cortices affected face processing, such that reduced integrity was related to poorer performance. However, in the present study, we did not collect data on the integrity of the white matter tracts and thus could not test this relationship with respect to speed in facial emotion identification. As communication between regions may play a vital role in these structure-function relationships, it will be important in future research to examine the extent to which age differences in structural and/or functional connectivity influence performance on facial emotion processing across the adult lifespan.

Interestingly, research has shown that childhood cognitive ability predicts the relationship between cognitive ability and structural brain integrity at older ages (Karama et al., 2014). This suggests that there may be a lifelong association between the preservation of brain structures and successful cognitive aging. It is important to highlight that there is a wide range of inter- and intra-individual variability in aging across structural, functional and behavioral measures. While both cross-sectional and longitudinal studies generally show atrophy of brain structures with age (Fotenos et al., 2005), cross-sectional comparisons of cognition generally reveal decline, while longitudinal comparisons generally reveal stability (Salthouse, 2014). Moreover, longitudinal studies demonstrate that, in healthy older adults, changes in brain volume are not uniform across regions, nor are they uniform across individuals (Resnick et al., 2003; Raz et al., 2005). Factors such as vascular health (e.g., hypertension), genetics (e.g., apolipoprotein E genotype), cognitive reserve, exercise and social interaction can all affect brain aging, both positively and negatively (Moffat et al., 2000; Raz et al., 2005; Stern, 2009; Hayes et al., 2014; Love et al., 2015), which can in turn affect functional activation and behavioral performance outcomes. Some of these factors are modifiable, so properly managing them can help to reduce age-related changes. This is particularly important, as, often times, research studies, including the present one, are limited by cross-sectional designs that only capture a snapshot of a participant’s general functioning and well-being. This highlights the importance for future research to use longitudinal data to determine neuroimaging markers of age-related cognitive change. It may be the case that the older adults in our study with larger hippocampal volumes have always done well with the identification of facial emotions.

Some study limitations need to be considered when interpreting our findings. The results of this study are preliminary in nature and replication from an independent sample is warranted. There are limited data available for the neuroanatomical basis of facial emotion identification and, even though the present study’s results are preliminary in nature, they are important for the investigation of this topic to progress. In addition, the relatively high accuracy in our older adult sample (and no age group differences in accuracy) may reflect the overall high functional level of the older participants recruited into our study. The facial emotion identification task in our study employed a simple response scheme (i.e., press of one of three keys to indicate one of three distinct facial expressions) and only investigated one positive and one negative facial expression. The relatively low demand on cognitive, perceptual and emotional capacities associated with the current task may have resulted in relatively reduced age-related behavioral impairments.

While we observed significant age interactions in the effect of hippocampus volume on task performance, we did not find effects in the other predicted ROIs (i.e., anterior cingulate cortex, fusiform gyrus, insula and amygdala). The relationship between brain structure and function is not well-understood and may explain our lack of findings in some of the predicted regions. For example, we looked at volume of the fusiform gyrus and not of the fusiform face area, which is a specific region of the fusiform gyrus involved in face processing (McCarthy et al., 1997). It may be that global volume of the fusiform gyrus is not involved in face processing. Future directions for research in facial emotion identification should include the use of a functional localizer task to individually identify ROIs, which could then be targeted for volume extraction.

Studies have demonstrated that gray matter volume in brain regions that are important for identification of facial emotions can be increased via cognitive training (Engvig et al., 2014), erobic exercise (Gondoh et al., 2009; Killgore et al., 2013) and mindful meditation (Hölzel et al., 2011; Santarnecchi et al., 2014). It will be informative to examine the extent to which engaging in these activities may help to counteract age-related atrophy in the brain, thereby helping to improve face and emotion processing in aging, given the promising evidence from our data that greater hippocampal volume predicted faster correct facial emotion identification in older adults.

In conclusion, our findings add to a research gap on the effects of regional brain volumes on facial emotion identification in aging. Overall, aging is characterized by reductions in gray matter volumes. However, there is support for the general notion that “bigger brain volume is better” (Eyler and Kovacevic, 2010; Bickart et al., 2011; but see Tottenham et al., 2010). Our results are consistent with this notion, in that we find that larger regional volumes of task-relevant regions predicted improved performance in a task related to face and emotion processing in advanced age. We hope that findings from the current study will spur additional research on this topic.

## Author Contributions

NCE and HF designed the study and acquired the data. JP processed the neuroimaging data. SMS and TL performed the statistical analyses. All authors contributed to data interpretation and to the writing of the article.

## Funding

This work was supported by the Swedish Research Council (2008-2356 to HF); Konung Gustaf V:s och Drottning Victorias Frimurarstiftelse (to HF), and the National Institute on Aging (T32 AG020499-11 to SMS). NCE received partial salary support from the Center for Cognitive Aging and Memory at the University of Florida.

## Conflict of Interest Statement

The authors declare that the research was conducted in the absence of any commercial or financial relationships that could be construed as a potential conflict of interest.

## Footnotes

^1^http://surfer.nmr.mgh.harvard.edu/
